# Supplementary material for: Biologically transparent illumination is a safe, fast, and simple technique for detecting the correct position of the nasogastric tube in surgical patients under general anesthesia
Source: PLoS One. 2021 Apr 29;16(4):e0250258. doi: 10.1371/journal.pone.0250258 (PMC8084215; doi:10.1371/journal.pone.0250258)
Supplement: Attachment — Submitted filename: Response to Reviewers.docx [file pone.0250258.s002.docx]

Response to Reviewers

***Comments to the Author***

*1. Is the manuscript technically sound, and do the data support the conclusions?
The manuscript must describe a technically sound piece of scientific research with data that supports the conclusions. Experiments must have been conducted rigorously, with appropriate controls, replication, and sample sizes. The conclusions must be drawn appropriately based on the data presented.*

*Reviewer #1: Yes*

*Reviewer #2: Yes*

Reply: Thank you very much.

*2. Has the statistical analysis been performed appropriately and rigorously?*

*Reviewer #1: Yes*

*Reviewer #2: Yes*

Reply: Thank you very much

*3. Have the authors made all data underlying the findings in their manuscript fully available?

The* [*PLOS Data policy*](http://www.plosone.org/static/policies.action#sharing) *requires authors to make all data underlying the findings described in their manuscript fully available without restriction, with rare exception (please refer to the Data Availability Statement in the manuscript PDF file). The data should be provided as part of the manuscript or its supporting information, or deposited to a public repository. For example, in addition to summary statistics, the data points behind means, medians and variance measures should be available. If there are restrictions on publicly sharing data—e.g. participant privacy or use of data from a third party—those must be specified.*

*Reviewer #1: No*

*Reviewer #2: Yes*

Reply: We added the additional data about the obese patients, and the statement “We will open all data underlying the findings described in the present study without restriction according to the request.” in the revised manuscript.

*4. Is the manuscript presented in an intelligible fashion and written in standard English?

PLOS ONE does not copyedit accepted manuscripts, so the language in submitted articles must be clear, correct, and unambiguous. Any typographical or grammatical errors should be corrected at revision, so please note any specific errors here.*

*Reviewer #1: Yes*

*Reviewer #2: Yes*

Reply: Thank you very much.

*5. Review Comments to the Author

Please use the space provided to explain your answers to the questions above. You may also include additional comments for the author, including concerns about dual publication, research ethics, or publication ethics. (Please upload your review as an attachment if it exceeds 20,000 characters)*

*Reviewer #1: The paper would benefit from including data to support the conclusions made, including the % of obese women in the cohort; stratification of test performance by obesity; an evaluation of how BT sensitivity could be improved by combining it with auscultation and gastric pH (other point of care tools to ascertain NGT location); and a breakdown of + BT Light by anatomical site. I would recommend publication once these issues are addressed.*

Reply:

According to the reviewer’s comments, we added the % obese patients in the cohort and offered the sensitivity and specificity of BT-light in the obese patients in the Material and Methods, Result and Discussion sections (Page 7, 11, 13-14 Table 1 and 3).

The purpose of this study is to identify the effectiveness of BT light to detect the correct position of NGT in the stomach. Therefore, we did not analyze the combined effect of each test. In general, combining of different kinds of test provides not only advantages but also disadvantages which are involved in each test. Actually, the cases of true positive (92 cases) are increased by combining of BT light, auscultation and identification of gastric aspirate tests (the positive sign of the combination technique is defined as when any of three techniques demonstrating the positive sign) in this study, but those of false positive (4 cases) are also increased compared to the BT light itself. The sensitivity of combination of three test is 99 % (92/93) which is much higher than that of the BT light itself as expected by reviewer. On the other hand, the specificity of combination of three tests is 56 % (5/9) which is much lower than that of the BT light itself. Consequently, X-ray examination is needed in the BT light negative cases to secure safety of NGT use even when auscultation and identification of gastric aspirate tests showing positive sign.

We did not categorize anatomical site of epigastric area. When BT light was identified in any epigastric area, we judged it positive sigh of BT light. We are sorry for the incomprehensible caption of Fig 2. We changed the caption of Fig. 2 (Page 10).

*Minor comments include:

Page 6 – define ‘with or without finger pressure’. Where and how this is done?*

Reply: As shown in Fig 2, the pressure was applied with the index finger at 3-4 cm below the tenth rib on the midclavicular line in epigastric area. This comment is added in the Methods section (Page 6-7).

*Page 7 – ‘on the basis that the lower limited of the 95% confidence interval exceeds 95%’.. please clarify exceeds 95% of what?*

Reply: We are sorry for incomplete phrase. The lower limited of the 95% confidence interval exceeds 95% of positive predictive value. This phrase is added in the Materials and Methods (Page 8)  *Page 8 – Table 1. Can you provide range and % of overweight and obese patients in the cohort. One would expect performance BTL to suffer the more obese the patients are.*

Reply: According to the reviewer’s comments, we added the % obese patients in Table 1 (Page 9).

*Page 8: ‘the power of BT light to identify the correct position of the NGT was not equivalent to that of X-ray examination’ – can you rephrase this? It’s not entirely clear what is referred to here.*

Reply: As the reviewer pointed out, this sentence is incomprehensible. We changed the sentence (Page 10). *Page 9: Remove ‘ the latter of which was quite low’ and expand on this in the discussion.*

Reply: We deleted *‘ the latter of which was quite low’* and discussed about the low specificity in the Discussion section (Page 14).  *Page 9: Figure 2. This figure caption mentions anatomical areas where BT light was detected. Is there a breakdown of this available per anatomical site, and how this affected test performance? You are alluding to this in the first paragraph of your discussion but you are not showing the data.*

Reply: We did not categorize anatomical site of epigastric area. When BT light was identified in any epigastric area, we judged it positive sigh of BT light. We are sorry for the incomprehensible caption of Fig 2. We changed the caption of Fig 2 (Page 10). We think there is no breakdown of anatomical site of epigastric area. Therefore, the breakdown did not affect test performance. *Abdominal wall = epigastric area? Use consistent descriptors for your anatomical sites in the paper.*

Reply: As the reviewer pointed out, we used consistent descriptors for our anatomical sites in the paper.

*Page 11: I suggest to move the section on BTL and abdominal wall thickness to the results part of the paper, including methodology of the measurements. As above, include more details of the proportion of obese patients etc in your results. Potentially stratify results BMI.*

Reply: As the reviewer pointed out, we moved the section on BTL and abdominal wall thickness to the results part of the paper and added more details of the proportion of obese patients in the Results section (Page 10-11).

*Page 13 – discuss safety of BT light (here or in background section)*

Reply: We added discussion of safety of BT light here (Page 12)

*Page 12 – can a combination of BT, auscultation, and gastric aspirate (can be done in at bedside) be used to improve sensitivity?*

Reply: Yes, by the combining the three techniques, the sensitivity is improved, however, the specificity is deteriorated.

*Reviewer #2: I think it’s a clever approach.maybe more patients could have been found.
Nicely presented.Helpful for clinical practice.Simple techniques.Also nice to see the pictures at the end.
I have no more remarks about it*

Reply: We are so appreciated.

*6. PLOS authors have the option to publish the peer review history of their article (*[*what does this mean?*](https://journals.plos.org/plosone/s/editorial-and-peer-review-process#loc-peer-review-history)*). If published, this will include your full peer review and any attached files.
If you choose “no”, your identity will remain anonymous but your review may still be made public.*
***Do you want your identity to be public for this peer review?*** *For information about this choice, including consent withdrawal, please see our* [*Privacy Policy*](https://www.plos.org/privacy-policy)*.*

*Reviewer #1: No*

*Reviewer #2: No*

Reply: I have no comment.
