# Supplementary material for: Biologically transparent illumination is a safe, fast, and simple technique for detecting the correct position of the nasogastric tube in surgical patients under general anesthesia
Source: PLoS One. 2021 Apr 29;16(4):e0250258. doi: 10.1371/journal.pone.0250258 (PMC8084215; doi:10.1371/journal.pone.0250258)
Supplement: Attachment — Submitted filename: Response to the Editor.docx [file pone.0250258.s003.docx]

Reply to the Editor.

*Journal Requirements:*

*Please review your reference list to ensure that it is complete and correct. If you have cited papers that have been retracted, please include the rationale for doing so in the manuscript text, or remove these references and replace them with relevant current references. Any changes to the reference list should be mentioned in the rebuttal letter that accompanies your revised manuscript. If you need to cite a retracted article, indicate the article’s retracted status in the References list and also include a citation and full reference for the retraction notice.*

We reviewed all the references in our manuscript. We can not find any retracted papers in our references. If you have any information about the retracted paper in our references, please let me know.

*Additional Editor Comments (if provided):

On page 5 You have added the sentence: "We will open all data underlying the findings described in the present study without restriction according to the request" and this sentence is suitable for a reply to the reviewers, but should not be added to the manuscript. Kindly include all underlying data never the less.*

According to the pointing out of the Editor, we delete the sentence (page 5). All underling data are included in the manuscript.

*Kindly have a native English speaker revise the introduced changes, the language in most cases will require some modification. Specifically reviewer 1 had earlier asked to revise the sentence on page 10, which you kindly revised to: "Because the sensitivity for verifying the correct position of the NTG with BT light did not close to 100% when X-ray examination is used as the reference standard, the usefulness of BT light to identify the correct position of the NGT was not equivalent to that of X-ray examination." Unfortunately this sentence is still hard to understand, kindly revise together with a native English speaker.*

 We are apologize for our limited fluency in English. The manuscript has been professionally edited by a native English speaker familiar with this area of research. The sentence *"Because the sensitivity for verifying the correct position of the NTG with BT light did not close to 100% when X-ray examination is used as the reference standard, the usefulness of BT light to identify the correct position of the NGT was not equivalent to that of X-ray examination."* was revised to “Because the sensitivity for detecting the correct position of the NTG with BT light was not close to 100% when X-ray examination was used as the reference standard, the usefulness of BT light in identifying the correct position of the NGT was not considered equivalent to that of X-ray examination. (Page 10).

The certification of the editing is enclosed.

Additional correction.

We are deeply sorry, but we found the simple mistakes in Table 2. The numbers of patients in Auscultation test were wrong (accordingly, sensitivity, and NPV). We corrected the numbers. Those corrections did not change any other contents in the manuscript including the conclusions at all. We are sorry again.
